# Emergence and characterization of a novel ST627-KL8 carbapenem-resistant *Klebsiella pneumoniae* lineage associated with ICU transmission in a tertiary hospital, China

**DOI:** 10.3389/fmicb.2025.1723336

**Published:** 2026-02-04

**Authors:** Xiaoxiao Pang, Guoxin Xu, Yu Zheng, Chao Ding, Wei Zhang, Hong Du, Long Chen

**Affiliations:** 1 Department of Clinical Laboratory, The First People’s Hospital of Zhangjiagang City, Zhangjiagang Hospital Affiliated to Soochow University, Zhangjiagang, China; 2 College of Veterinary Medicine, Nanjing Agricultural University, Nanjing, China; 3 Department of Clinical Laboratory, The Second Affiliated Hospital of Soochow University, Suzhou, China

**Keywords:** carbapenem-resistance, IncFII_K34_ plasmid, *Klebsiella pneumoniae*, nosocomial infection, ST627-KL8 type

## Abstract

**Introduction:**

Carbapenem-resistant *Klebsiella pneumoniae* (CRKP) poses a serious threat to public health. We characterized a rarely reported ST627-KL8 CRKP lineage associated with intensive care unit (ICU) transmission.

**Methods:**

Three isolates (ZJG29565, ZJG30140, and ZJG30146) were obtained from three patients in the ICU and subjected to antimicrobial susceptibility testing. Whole-genome sequencing (WGS) was performed to determine genomic characteristics, phylogenetic relationships, and plasmid content, followed by assessments of mucoviscosity, capsule quantification, serum resistance, and bacterial virulence using a *Galleria mellonella* (*G. mellonella*) infection model. Additionally, bacterial capsule morphology was observed via transmission electron microscopy (TEM).

**Results:**

SNP analysis (≤ 5 SNPs) confirmed clonal transmission within the ICU. Phylogenetic analysis placed ST627-KL8 as a distinct lineage closely related to ST14. All isolates carried an IncFII_K34_ plasmid encoding *bla*_KPC–2_, consistent with their carbapenem-resistant phenotype. Phenotypic assays—including TEM, mucoviscosity testing, serum resistance, and uronic acid quantification—demonstrated a thinner capsule and reduced mucoviscosity compared with the KL2 reference strain. In the *Galleria mellonella* model, ST627-KL8 exhibited intermediate virulence (66.7%–76.7% survival), between the hypervirulent *K. pneumoniae* ATCC 43816 strain (30.0%) and the low-virulence *K. pneumoniae* ATCC 700603 strain (96.7%).

**Discussion:**

This study identified a novel ST627-KL8 CRKP clone with intermediate virulence, consistent with its reduced capsule phenotype and lack of classical hypervirulence genes. These features, together with the subtle clinical presentations, may contribute to reduced clinical vigilance and delayed optimization of antimicrobial therapy. Importantly, ST627-KL8 CRKP carried the IncFII_K34_ *bla*_KPC–2_ plasmid, which has been reported to exhibit high conjugation frequency, posing a significant challenge in clinical settings.

## 1 Introduction

*Klebsiella pneumoniae* (*K. pneumoniae*), a gram-negative opportunistic pathogen, has emerged as a critical public health threat because of its concurrent evolution toward enhanced virulence and antimicrobial resistance. As a predominant causative agent of hospital-acquired infections (HAIs), *K. pneumoniae* is implicated in pneumonia, bloodstream infections (BSIs), urinary tract infections (UTIs), and intra-abdominal infections, with high mortality rates among immunocompromised patients (Wyres et al., 2020; Quan et al., 2021; Holmes et al., 2025). Cephalosporins and carbapenems remain the primary therapeutic options for infections caused by ESBL-producing *K. pneumoniae* (Escolà-Vergé et al., 2019; Dequidt et al., 2023). However, the rapid dissemination of carbapenem-resistant *K. pneumoniae* (CRKP), fueled by the global overuse of carbapenems, has rendered these infections progressively harder to manage (Wang et al., 2024; Zhang et al., 2019). Surveillance data from the CHINET Antimicrobial Resistance Surveillance Network indicate a marked increase in carbapenem resistance among CRKP in China: resistance to imipenem and meropenem rose from 3.0% and 2.9% in 2005 to 22.6% and 23.4% in 2024, respectively (Guo et al., 2025).

In China, the KL47 and KL64 capsular serotypes represent the most prevalent CRKP lineages, with > 90% belonging to the pandemic ST11 clone. These strains frequently harbor *bla*_KPC–2_ carbapenemase genes, driving their dual threat of hypervirulence and multidrug resistance. Additional serotypes, including KL19, KL25, and KL10, contribute to regional outbreaks but lack the epidemiological dominance of KL47/KL64-ST11 hybrids (Liu et al., 2018; Li et al., 2020; Hu et al., 2024). While global CRKP research has focused on high-prevalence clones (e.g., ST11, ST258) and virulent serotypes (e.g., KL1, KL2), other important epidemic sequence types include ST15, ST65, ST37, and ST307, among others (Zhu et al., 2016, 2024; Liu C. et al., 2022; Yang et al., 2013; Feng et al., 2023; Palmieri et al., 2019). Although significant progress has been made in the epidemiological studies of CRKP worldwide, the majority of existing research findings focus on the aforementioned capsular serotypes and ST typing. At the same time, the rare ST627 type has received minimal attention. The ST627 lineage of *K. pneumoniae* has been sporadically reported in clinical settings, with several isolates documented to date. These include four KL24 serotype strains isolated from a bloodstream infection in Egypt (ST627-KL24) (Enany et al., 2022) and a carbapenem-resistant variant harboring *bla*_KPC–4_ within the Tn4401b transposon in South Korea (Yoon et al., 2018). Although reports of ST627 are scarce, the few geographically dispersed and epidemiologically unrelated cases suggest potential cryptic spread and highlight gaps in our understanding of its evolutionary origins.

In this study, motivated by a surveillance initiative to understand the local distribution of *K. pneumoniae* serotypes, we retrospectively investigated a small-scale ICU outbreak that occurred over a 46-day period from April to June 2018 at a tertiary hospital in China and was caused by a rare ST627-KL8 clone. This study aims to molecularly and clinically characterize the involved ST627-KL8 *K. pneumoniae* clone, which harbored the *bla*_KPC–2_ carbapenemase gene. Our work calls for increased vigilance against such under-surveilled clones to mitigate future outbreaks.

## 2 Materials and methods

### 2.1 Isolation of bacterial strains

Bacterial strains ZJG29565, ZJG30140, and ZJG30146 were isolated from sputum or blood culture specimens obtained from three patients in the ICU. The isolates were identified via MALDI-TOF MS (Bruker Microflex LT-SH, Bruker Daltonik GmbH, Germany). Reference strains *K. pneumoniae* ATCC 43816 and *K. pneumoniae* ATCC 700603 were stored in our laboratory.

### 2.2 Antibiotic susceptibility testing and carbapenemase detection

The antimicrobial susceptibility tests of bacterial strains ZJG29565, ZJG30140, and ZJG30146 were performed using the standard broth microdilution method (Morris et al., 2020), and the results were interpreted according to the 2025 Clinical and Laboratory Standards Institute guidelines (CLSI, 2025). Antibiotic susceptibility testing was performed using the VITEK-2 Compact system (bioMérieux, France). All experimental procedures were conducted with three independent biological replicates, prepared on separate dates.

The carbapenem inactivation method (mCIM) and EDTA carbapenem inactivation method (eCIM) were performed to detect carbapenemase according to CLSI guidelines (CLSI, 2025). *K. pneumoniae* ATCC BAA-1705 and *K. pneumoniae* ATCC BAA-2146 were used as positive quality control strains; *Escherichia coli* ATCC 25922 was used as a negative quality control strain. For each isolate and control strain, a 1-μL loopful of bacteria from an overnight blood agar plate was suspended in 2 mL tryptic soy broth (TSB) and vortexed for 10–15 s. For mCIM, a meropenem disk was added directly to the suspension. For eCIM, 20 μL of 0.5 M EDTA was added before introducing the meropenem disk. After 4 h of incubation at 37 °C, the disks were retrieved and placed on Mueller-Hinton agar plates freshly lawned with the *E. coli* ATCC 25922 indicator strain. Zones of inhibition were measured after 18 h of incubation, and results were interpreted according to CLSI criteria.

### 2.3 Whole-genome sequencing analysis

Genomic DNA was extracted from each isolate using the Solarbio Bacterial Genomic DNA Extraction Kit (Beijing, China) and sequenced on an Illumina X Plus platform (San Diego, CA, USA). Two of the isolates, ZJG29565 and ZJG30146, were selected for long-read sequencing on the PacBio Sequel system, yielding 547,144 and 701,921 raw reads, respectively. The raw FASTQ reads underwent quality filtering (Q-score ≥ 20) and automatic adapter trimming. *De novo* genome assembly was performed using the SPAdes (v3.5.0) or Canu (v1.3) (Bankevich et al., 2012; Koren et al., 2017). The detection of single-nucleotide polymorphisms (SNPs) in three isolates was performed with Snippy 4.6.0. Multi-locus sequence typing (MLST), serotype, and virulence factor profiling were performed for the three clinical isolates and reference strains using Kleborate v3 (Comandatore et al., 2019) and VFDB (Liu B. et al., 2022). The existence of *peg-344* was identified by Blast. Antibiotic resistance genes were searched using Kleborate v3. The plasmid type carried by the strain was identified using pMLST and plasmidFinder (Carattoli et al., 2014; Ruzickova et al., 2025).

### 2.4 Phylogenetic tree and comparative genomic analysis

BV-BRC Similar Genome Finder was used to identify closely related public *K. pneumoniae* genomes. Core single-nucleotide polymorphisms (SNPs) were identified using kSNP 3.0 (Gardner et al., 2015), and a maximum-likelihood tree was inferred using FastTree, followed by visualization and annotation in iTOL v7 (Letunic and Bork, 2021). Core-genome multilocus sequence typing (cgMLST) was performed in Ridom SeqSphere+ using the *K. pneumoniae* cgMLST scheme for 61 isolates, including our ST627-KL8 strains and related public genomes. A minimum spanning tree was generated from the allelic distance matrix, with nodes colored by sequence type (ST) and clonal complexes (CCs) annotated according to the clustering patterns. Comparative genomic analysis of chromosomal and plasmid architectures was conducted using BRIG v0.95 (Alikhan et al., 2011) to visualize sequence homology and structural variations against the reference genome. The sequence of ZJG29565 was used as the reference. Capsular gene cluster synteny comparison between the three clinical isolates and some prevalent serotypes was analyzed using Easyfig v2.2.3 (Sullivan et al., 2011).

### 2.5 Transmission electron microscopy (TEM)

Four distinct K-type *K. pneumoniae* strains were inoculated onto blood agar plates and incubated overnight at 37 °C. The subsequent day, bacterial colonies were harvested and subjected to fixation with 2.5% glutaraldehyde (Solarbio) for 12 h. The prepared samples were observed using a Hitachi transmission electron microscope system operating at 80.0 kV in high-contrast mode. For each isolate, a single bacterium with a distinct cell boundary was randomly selected and imaged at 20,000× magnification for analysis.

### 2.6 Mucoviscosity and capsule quantification

The mucoviscosity of strains was determined by a semi-quantitative measurement as previously described (Palacios et al., 2018). Briefly, each strain was cultured in LB overnight and then diluted to 1 × 10^9^ CFU in 1 mL LB in a microcentrifuge tube. After being centrifuged at 1,000 *g* for 5 min, the supernatant from each tube was transferred into 96-well plates. The mucoviscosity levels were determined by calculating the ratio of the OD_600_ value of the supernatant to the OD_600_ value of the original culture. To quantify the capsule level of each strain, the uronic acid was extracted and quantified as described previously (Wang et al., 2023). In brief, 5 × 10^8^ CFU of bacteria in 500 μL PBS was mixed with 100 μL 1% Zwittergent 3–14 detergent in 100 mM citric acid. After being heated for 20 min at 50°C and centrifuged at 13,000 *g* for 5 min, the supernatant from the samples was transferred into tubes and mixed with 1.2 mL ethanol and centrifuged. The pellet was dried and resuspended in 200 μL sterile water, and then 1.2 mL of tetraborate solution (12.5 mM sodium tetraborate in sulfuric acid) was added. The reaction mixture was vortexed and incubated at 100°C for 5 min and then immediately placed on ice for 10 min. After the addition of 20 μL hydroxyphenyl reagent, the reaction mixture was incubated at room temperature for 5 min and the OD_520_ value was measured. The uronic acid was determined by calculating the ratio of the OD_520_ value to the OD_600_ value of the original culture.

### 2.7 Serum resistance

The serum resistance ability of the strains was determined using the serum killing assay as previously described (Hu et al., 2024), with small modifications. Briefly, 50 μL of 1 × 10^6^ CFU/mL bacteria in PBS was mixed with 150 μL sera from healthy volunteers. After 0, 1, 2, and 3 h of incubation at 37°C, the mixture was diluted and then plated on LB agar plates. The bacterial colonies were counted, and the survival rate was calculated relative to 0 h.

### 2.8 *Galleria mellonella* infection model

Final-instar *Galleria mellonella* (*G. mellonella*) with body weights ranging from 190 to 220 mg were systematically selected to minimize inter-individual variability at baseline. It was implemented to ensure experimental consistency across all treatment cohorts. It was randomly allocated into seven experimental groups (*n* = 30 larvae per group) with three independent technical replicates per condition. Three clinical strains and two reference strains (*K. pneumoniae* ATCC 700603 and *K. pneumoniae* ATCC 43816) were cultivated in 1 mL of Luria-Bertani (LB) broth at 37 °C with shaking at 200 rpm overnight. A negative control group, consisting of physiological saline (PBS), and a blank control group were also used in the experiment. The following day, the cultures were diluted 200-fold with LB medium and incubated for 1–2 h under the same shaking conditions to achieve the logarithmic phase (∼10^8^ CFU/mL, 0.3 OD at 600 nm). The bacteria were then washed with PBS and adjusted to a concentration of approximately 10^6^ CFU/mL. Ultimately, 10 μL of the bacterial suspension (10^4^ CFU) was injected into the last left proleg of the *G. mellonella*. After infection, the number of survivors was recorded at 37 °C for seven consecutive days. Data are representative of three independent experiments.

### 2.9 Statistical analysis

GraphPad Prism software (version 6.0) was utilized for statistical analyses. Data from the three clinical ST627-KL8 *K. pneumoniae* isolates (ZJG29565, ZJG30140, ZJG30146), *K. pneumoniae* ATCC 43816 and *K. pneumoniae* ATCC 700603, are presented as the mean ± standard deviation (SD) from at least three independent replicates. To evaluate the differences among the groups, a one-way analysis of variance (ANOVA) was performed. This was followed by Dunnett’s *post-hoc* test for multiple comparisons. A *P*-value of less than 0.05 was considered statistically significant. Survival curves were plotted using the Kaplan–Meier method, and differences between groups were compared using the Log-rank test in GraphPad Prism software to determine virulence.

## 3 Results

### 3.1 Clinical information of three patients infected with CRKP

Through our retrospective analysis, we identified three rare ST627-KL8 type CRKP isolates, designated as ZJG29565, ZJG30140, and ZJG30146. These three isolates were recovered from sputum or blood specimens collected from hospitalized patients between April and June 2018.

Patient 1 (strain: ZJG29565), a 73-year-old male with a 10-year history of cerebral infarction, was admitted to the hospital due to a 4-h episode of altered consciousness. Upon initial examination, his blood glucose level was elevated at 12.9 mmol/L, and he presented with severe hypoxemia, indicated by an oxygen saturation of 40%. A chest radiograph revealed mild exudative changes in the left lung. Consequently, he was diagnosed with type II respiratory failure. The initial antibiotic regimen for pulmonary infection was Piperacillin-Sulbactam. A sputum culture 11 days later revealed multidrug-resistant *K. pneumoniae*. However, since the patient was afebrile with minimal airway secretions, contamination of the specimen during endotracheal intubation was suspected, and the original antibiotic regimen was maintained. On the day CRKP was detected, the patient was discharged at the family’s request while still requiring endotracheal intubation and mechanical ventilation.

Patient 2 (strain: ZJG30140), a 25-year-old male, was admitted for hemorrhagic shock after his lower extremities were crushed by a vehicle, with a blood pressure of 82/42 mmHg upon admission. He presented with bilateral thigh degloving injuries, with exposure of muscles, tendons, and bones, and severely compromised distal perfusion. He was admitted urgently and underwent surgery. The initial antibiotic protocol included broad-spectrum coverage with Piperacillin-Tazobactam and Vancomycin. Following the subsequent development of a CRKP bloodstream infection (bacteremia), the antibiotic regimen was escalated to Imipenem-Cilastatin combined with Tigecycline. After 12 days of this targeted treatment, CRKP was no longer detected in subsequent secretion cultures. The patient was subsequently transferred to a higher-level hospital for further management.

Patient 3 (strain: ZJG30146), a 69-year-old male, was admitted for a right femoral neck fracture sustained in a fall. Upon admission, his vital signs were within normal limits. The patient had a 5-year history of hypertension and gout, with renal dysfunction for six months. The patient developed severe pneumonia following a right total hip arthroplasty. The initial antibiotic, Cefoperazone-Sulbactam, was changed to Imipenem-Cilastatin (0.5 g q12h). Two days later, after CRKP was isolated from sputum culture, the regimen was further escalated to Imipenem-Cilastatin (1.0 g q8h) combined with Levofloxacin (0.5 g qd). The patient’s overall condition remained critical, and the family requested discharge and withdrawal of active treatment the following day.

**FIGURE 1:**
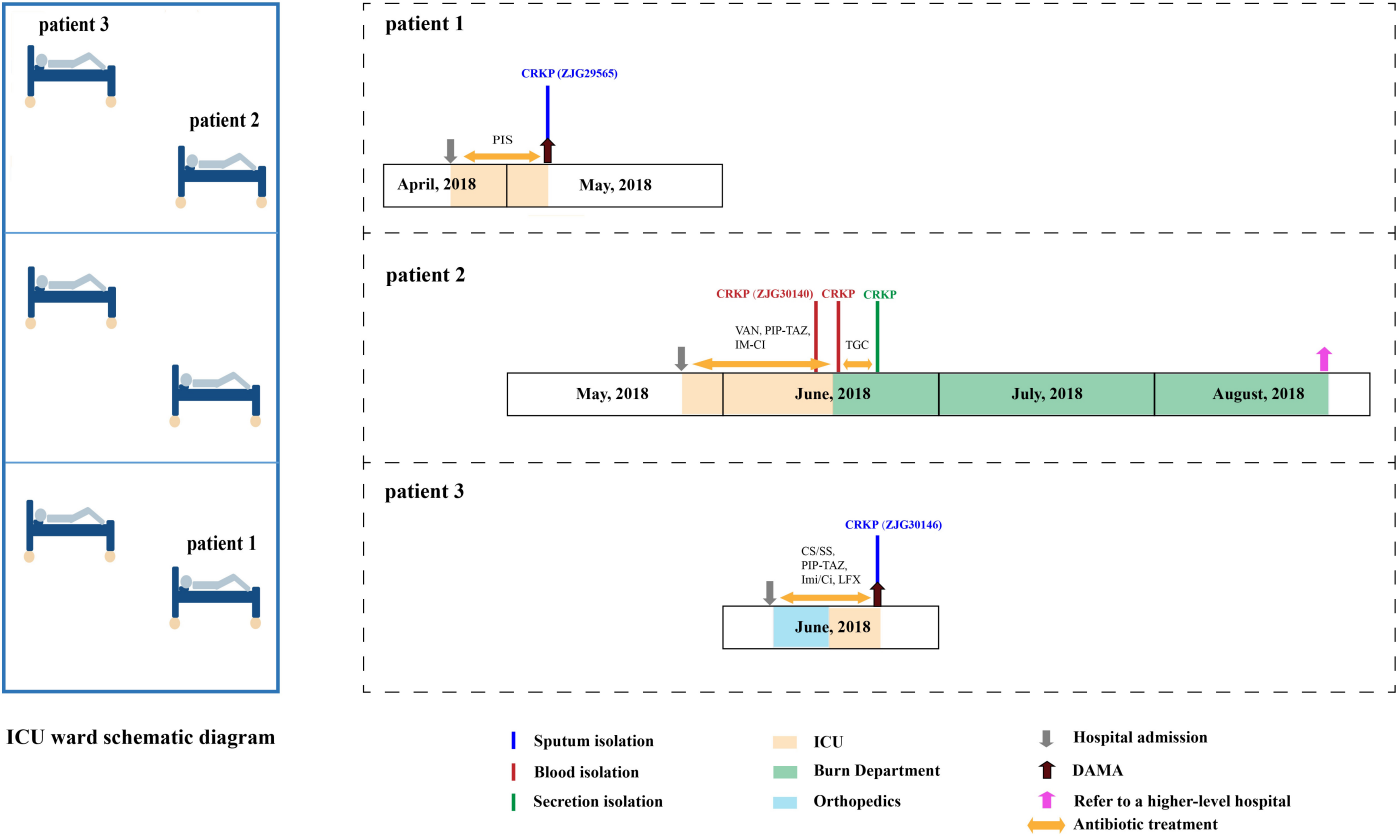
Epidemiology of the *K. pneumoniae* outbreak cases. Differently colored rectangles represent the duration of patient hospitalization in different wards or the duration of a strain infection. Bars of varying colors denote the different specimen types from which strains were isolated. Bidirectional arrows indicate antibiotic usage. DAMA indicates discharge against medical advice (withdrawal of active treatment by family request).

All three patients were admitted to the hospital and required ICU care between April and June 2018. ST627-KL8 CRKP was isolated from sputum cultures in patients 1 and 3, while patient 2 had ST627-KL8 CRKP detected in both blood and secretion cultures. Following treatment, patient 2 showed a favorable outcome and was transferred to a higher-level hospital for further treatment and correction of lower limb deformities. In contrast, Patient 1 was discharged against medical advice (DAMA) while still receiving endotracheal intubation and mechanical ventilation. Patient 3 likewise discontinued active treatment and was discharged by family request before completion of the planned therapy. These dispositions reflect family decisions made in the context of severe underlying illness and local care preferences, and do not in themselves constitute evidence that the ST627-KL8 infection directly caused a pathogen-driven adverse clinical outcome (Figure 1).

### 3.2 Antimicrobial susceptibility and carbapenemase detection

Three ST627-KL8 strains exhibited nearly identical antimicrobial susceptibility profiles (Magiorakos et al., 2012; Table 1). The antimicrobial susceptibility test results showed that these strains were resistant to more than half of the tested drugs. Apart from intrinsic resistance to certain antibiotics such as penicillins and some first-generation cephalosporins like cefalotin and ceftizoxime, three isolates were resistant to beta-lactam antibiotics, including imipenem, meropenem, and doripenem. However, the three isolates remained susceptible to aminoglycosides and quinolones. Additionally, mCIM and eCIM, the gold-standard phenotypic assays, were performed. All isolates were mCIM-positive and eCIM-negative. As such, the results are diagnostic of KPC-type serine carbapenemase production (Supplementary Figure 1).

**TABLE 1:** Antimicrobial susceptibility profiles of ST627-KL8 CRKP isolates.

Antibiotics	MIC (μg/mL)		
	ZJG29565	ZJG30140	ZJG30146
Amoxicillin/clavulanic acid	≥ 32	≥ 32	≥ 32
Amikacin	≤ 2	≤ 2	≤ 2
Aztreonam	16	16	16
Cefalotin	≥ 64	≥ 64	≥ 64
Ciprofloxacin	≤ 0.25	≤ 0.25	≤ 0.25
Cefpodoxime	≥ 8	≥ 8	≥ 8
Polymyxin B	≤ 0.5	≤ 0.5	≤ 0.5
Doxycycline	1	1	1
Imipenem	≥ 16	8	≥ 16
Levofloxacin	≤ 0.12	≤ 0.12	≤ 0.12
Meropenem	≥ 16	≥ 16	≥ 16
Minocycline	≤ 1	≤ 1	≤ 1
Moxifloxacin	≤ 0.25	≤ 0.25	≤ 0.25
Nalidixic acid	4	≤ 2	≤ 2
Norfloxacin	≤ 0.5	≤ 0.5	≤ 0.5
Piperacillin	≥ 128	≥ 128	≥ 128
Cefuroxime sodium	≥ 64	≥ 64	≥ 64
Cefuroxime axetil	≥ 64	≥ 64	≥ 64
Trimethoprim/ sulfamethoxazole	≤ 20	≤ 20	≤ 20
Cefotaxime	32	32	32
Ceftazidime	8	8	8
Ticarcillin/clavulanic acid	≥ 128	≥ 128	≥ 128
Tetracycline	≤ 1	≤ 1	≤ 1
Tobramycin	≤ 1	≤ 1	≤ 1
Piperacillin/ tazobactam	≥ 128	≥ 128	≥ 128
Teicoplanin	≤ 0.5	≤ 0.5	≤ 0.5
Cefoperazone/ sulbactam	16	16	16

### 3.3 Genome sequence of ST627-KL8 CRKP clinical isolates

**FIGURE 2:**
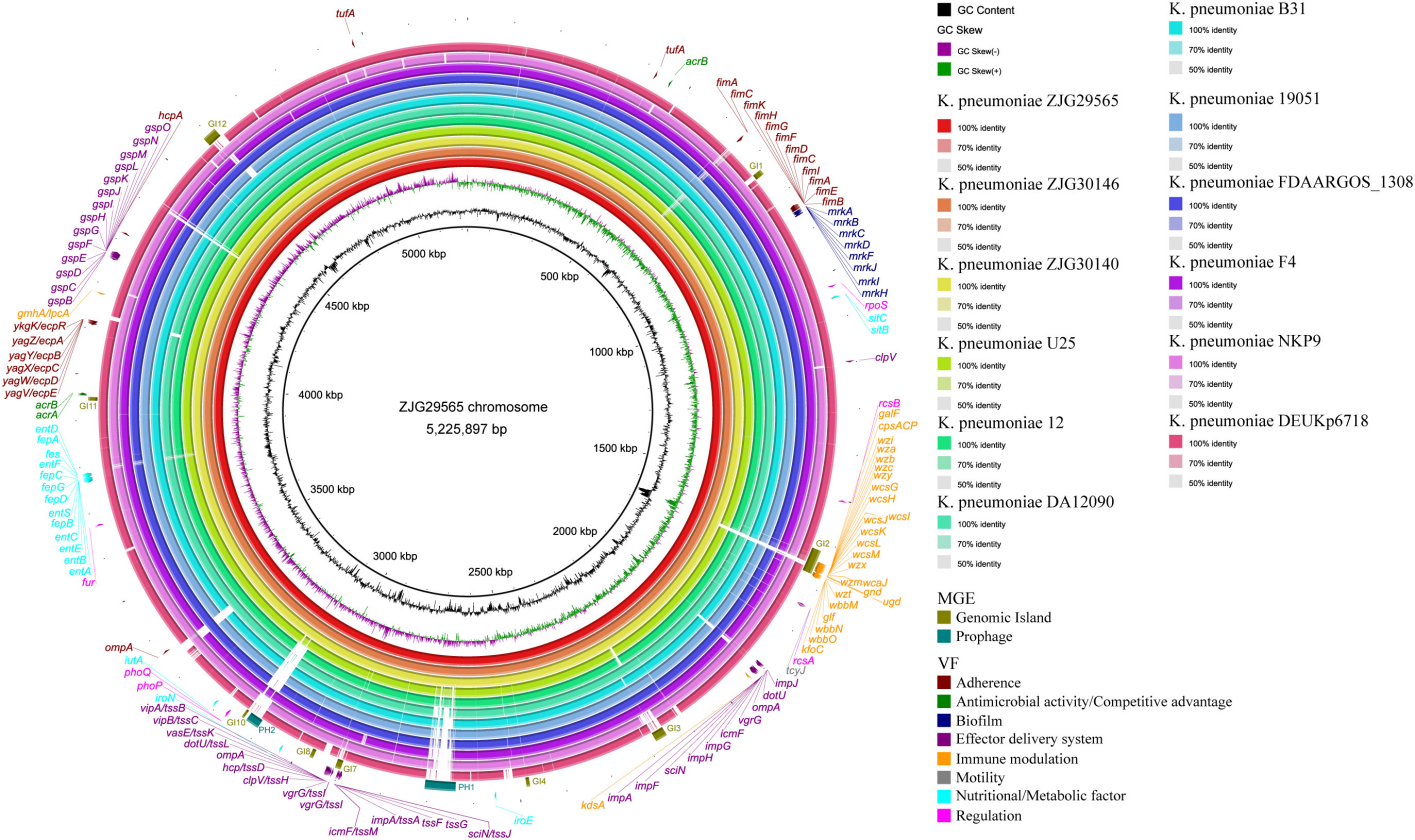
Schematic diagram of the whole genomes of three ST627-KL8 clinical isolates. The circles show (from inside to outside): strain ZJG29565, ZJG30146, ZJG30140, *K. pneumoniae* U25, *K. pneumoniae* 12, *K. pneumoniae* DA12090, *K. pneumoniae* B31, *K. pneumoniae* 19051, *K. pneumoniae* FDAARGOS_1308, *K. pneumoniae* F4, *K. pneumoniae* NKP9, *K. pneumoniae* DEUKp6718, GC skew [(G–C)/(G + C)], and GC content. MGE, mobile genetic elements; VF, virulence factors.

SNP analysis revealed 5 SNPs between ZJG30146 and ZJG29565, but none between ZJG30146 and ZJG30140 (Supplementary Table 1), supporting that the three isolates originated from a single clone. To obtain an overview of the genomic characteristics of ST627-KL8 CRKP isolates, we searched genomes similar to ZJG29565 through BV-BRC. We included the nine genomes most identical to ours in subsequent comparative genomic analysis (Supplementary Table 2). Comparative genomic analysis using BRIG v0.95 showed ZJG29565 had a full length of 5,225,897 bp and a GC content of 57.6%. ZJG30140 and ZJG30146 exhibited the highest similarity to ZJG29565, followed by *K. pneumoniae* U25 (CP012043), which showed 98% coverage and 99.91% sequence identity with ZJG29565 (Figure 2). Analysis of the whole-genome sequencing results using Kleborate and Blast revealed that all three strains carried only two resistance genes: *bla*_KPC–2_ and *bla*_SHV–115_. Additionally, they did not carry the classic virulence genes, such as *rmpA*, *rmpA2*, *iucABCD*, *iutA*, *peg-344*, or *iroBCDN* (Russo et al., 2025), which are typically associated with hypervirulent *K. pneumoniae*. We performed phylogenomic analysis to understand the evolutionary relationships and diversification of ST627-KL8 CRKP clinical isolates.

**FIGURE 3:**
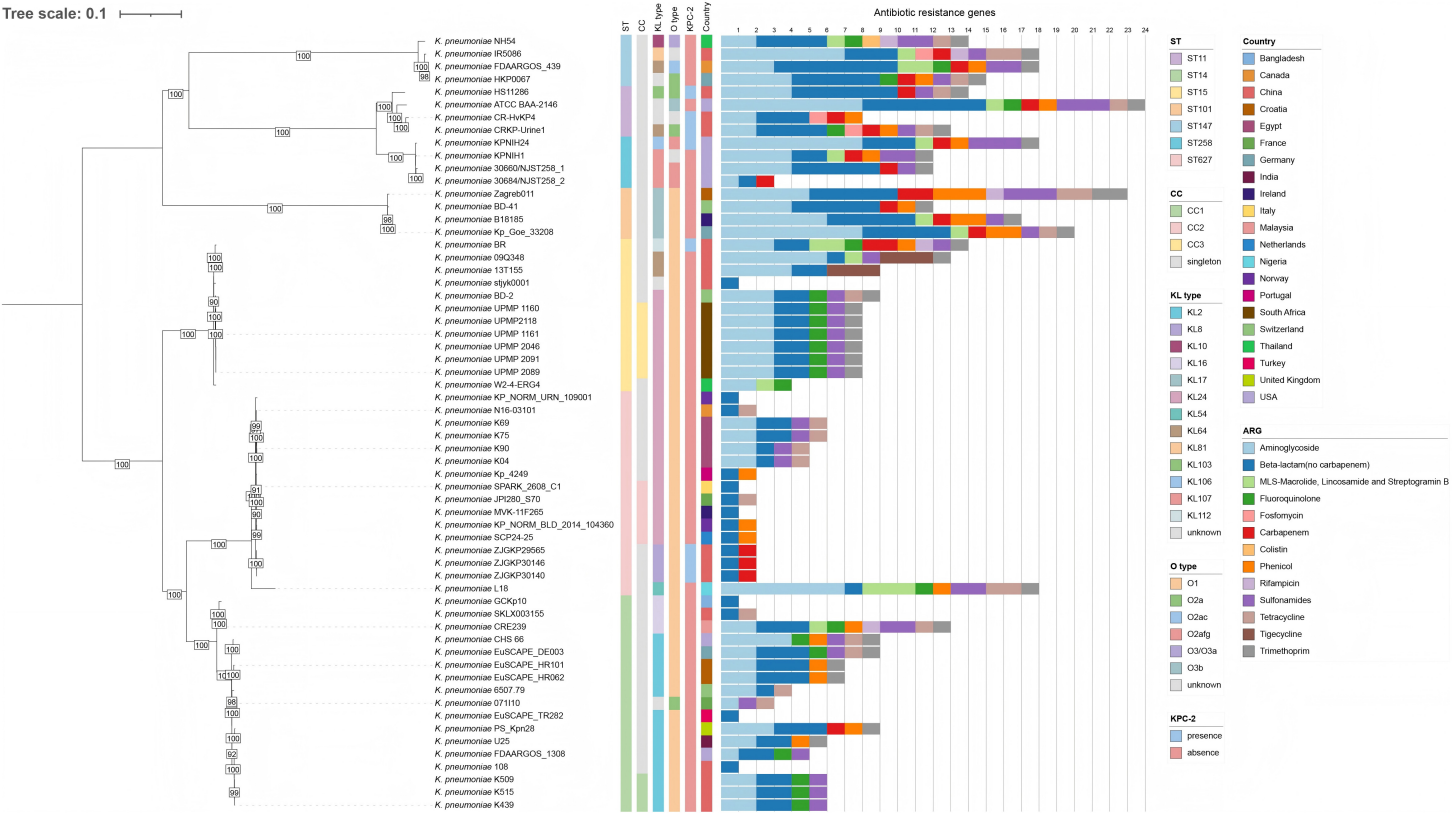
Core-genome SNP phylogeny and genomic characteristics of ST627 and related *K. pneumoniae* lineages. Maximum-likelihood phylogenetic tree based on core-genome SNPs of representative *K. pneumoniae* isolates, including the ST627-KL8 isolates from this study (indicated in bold). Branch support values (bootstrap percentages) are shown at the major nodes, and the scale bar indicates nucleotide substitutions per site. To the right of the tree, colored tracks denote sequence type (ST), clonal complex (CC), KL (capsular) locus type, O antigen (O) locus type, and country of origin, as indicated in the legends. Presence of *bla*_KPC–2_ is shown in a separate track (KPC-2). Antimicrobial resistance genes (ARGs) identified by Kleborate are summarized by resistance class and displayed as stacked bar plots (1–24) alongside each isolate, with colors indicating different ARG categories (e.g., aminoglycosides, beta-lactams, fluoroquinolones, carbapenems, colistin, tetracyclines, tigecycline, sulfonamides, trimethoprim, etc.). Together, the phylogeny and genomic annotations highlight the close relationship between ST627 and the ST14 clonal complex and the ARG profiles of the included high-risk lineages.

To place the ST627-KL8 isolates into a broader genomic context, we first used whole-genome similarity searches to identify the 29 *K. pneumoniae* genomes most closely related to our ST627-KL8 strains. This dataset was then supplemented with representative genomes of several major globally disseminated high-risk clones, including ST11, ST258, ST101 and ST147, as well as all publicly available ST627 genomes that met the quality criteria for core-genome SNP analysis (Figure 3 and Supplementary Table 3). Within the ST627 group, the three clinical isolates from this study clustered tightly together and formed a distinct sublineage. Apart from these three strains, which carried the KL8 capsular locus, most of the other ST627 genomes were associated with KL24, with a minority carrying KL54, indicating that the ST627-KL8 combination described here represents an unusual capsular variant within ST627. In the broader phylogenetic framework, ST627 isolates grouped within the ST14-associated clonal complex, whereas ST15 formed a separate cluster on a more distant branch. This pattern indicates that ST627 is more closely related to ST14 than to ST15 and is likely derived from an ST14-like ancestor. Collectively, these data support the conclusion that the ST627-KL8 CRKP isolates identified in this study belong to a novel lineage within the ST14-related *K. pneumoniae* population.

To further examine clonal relatedness, we performed core-genome MLST (cgMLST) using the Ridom SeqSphere+ scheme and generated a minimum spanning tree for 61 genomes (Supplementary Figure 2). Applying the ≤ 15-allele difference threshold used to define clonal complexes (CCs), three main CCs (CC1–CC3) were observed, each dominated by isolates of a single sequence type (CC1: ST14, CC2: ST15, CC3: ST627). Because substantial within-ST diversity exists, only subsets of ST14, ST15 and ST627 fell within these highlighted CCs, whereas the remaining isolates of each ST lay outside the shaded regions but still clustered closer to their own ST than to other STs. Notably, in the MST the ST627 cluster was positioned adjacent to ST14 isolates, indicating that ST627 shared the smallest cgMLST allelic distances with ST14 among the sequence types analyzed, in agreement with the core SNP phylogeny.

### 3.4 The IncFII_K34_ KPC-2 plasmid in ST627-KL8 CRKP clinical isolates

**FIGURE 4:**
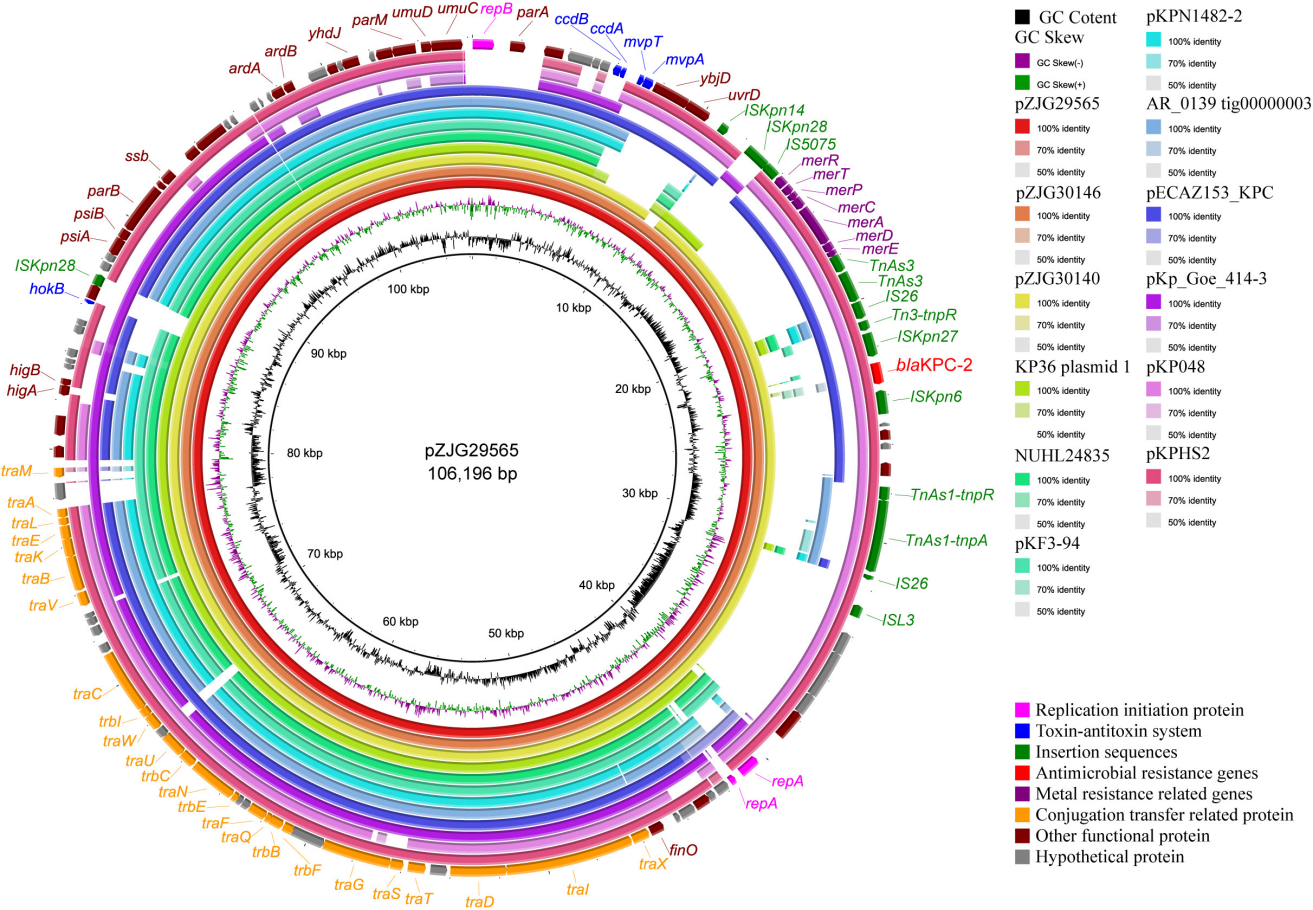
Schematic representation of IncFII_K34_ KPC-2 plasmid sequences from three ST627-KL8 clinical strains. The circles show (from inside to outside): plasmid pZJG29565, pZJG30146, pZJG30140, KP36 plasmid 1, NUHL24835 unnamed1, pKF3-94, pKPN1482-2, AR_0139 tig00000003, pECAZ153_KPC, pKp_Goe_414-3, pKP048, pKPHS2, GC skew [(G–C)/(G + C)], and GC content.

The sequencing results of Illumina and PacBio showed that ZJG29565 harbored an IncFII_K34_ plasmid carrying *bla*_KPC–2_, which was assigned as pZJG29565. Nine plasmids highly homologous to pZJG29565 were identified using BV-BRC and compared with plasmids from ST627-KL8 isolates (Supplementary Table 4). The structure comparison revealed that the plasmids carried by the three isolates were nearly identical in structure. The backbone of plasmid pZJG29565 was most similar to and shared 98.08% nucleotide identity and 90% coverage with an IncFII_K2_ plasmid pKPHS2, which was carried by a multidrug-resistant *K. pneumoniae* strain isolated in Shanghai (Liu et al., 2012; Figure 4).

### 3.5 Low mucoviscosity, intermediate serum resistance, reduced capsule production, and genomic features of the capsular polysaccharide locus in ST627-KL8 CRKP

**FIGURE 5:**
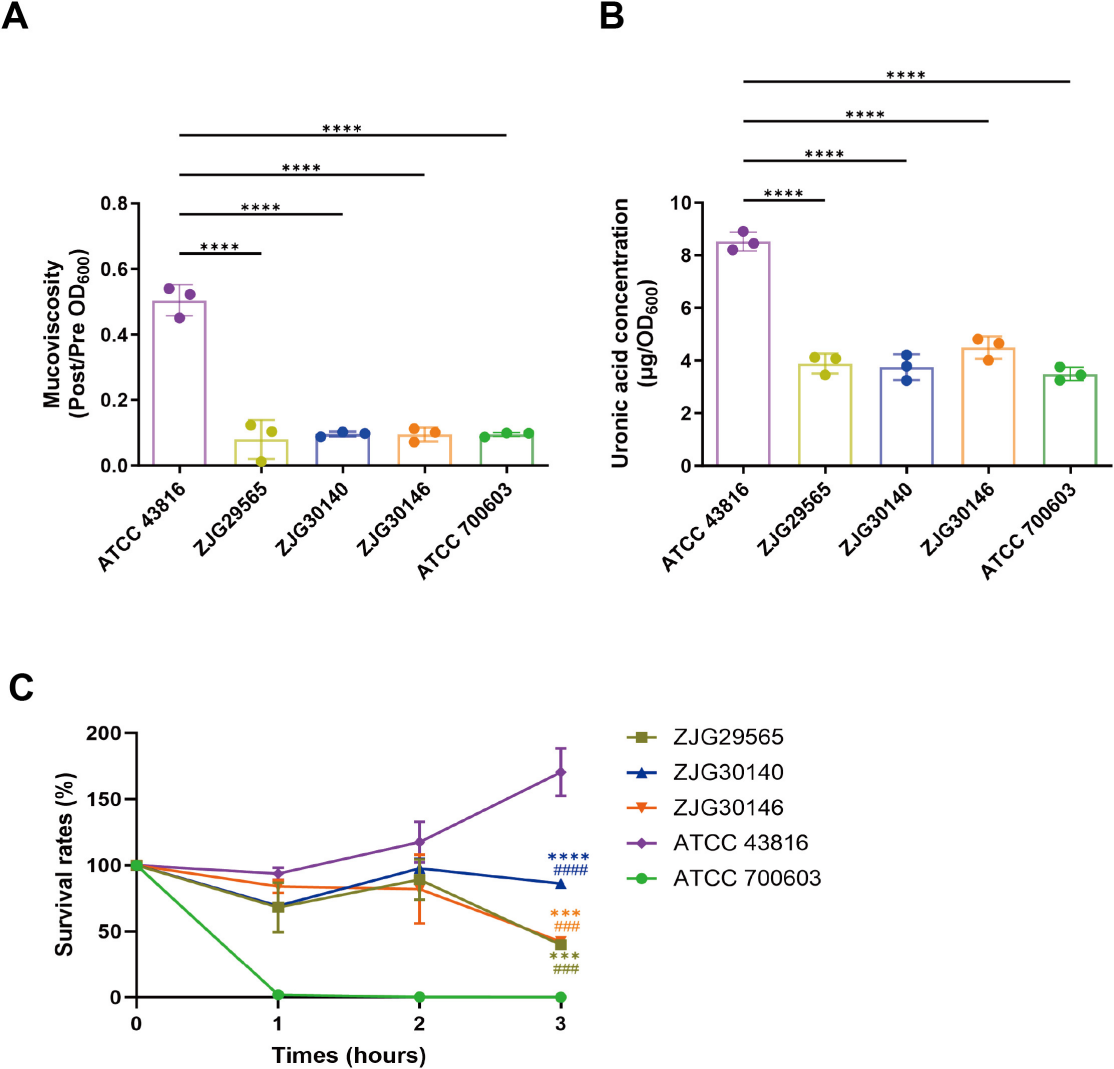
The mucoviscosity **(A)**, capsule quantification **(B)**, and serum resistance **(C)** of ST627-KL8 strains. **(A)** Mucoviscosity in three ST627-KL8 clinical strains, *K. pneumoniae* ATCC 43816 and *K. pneumoniae* ATCC 700603. The mucoviscosity of strains was determined by a semi-quantitative measurement. **(B)** Capsule uronic acid production in three ST627-KL8 clinical strains, *K. pneumoniae* ATCC 43816 and *K. pneumoniae* ATCC 700603. **(C)** The resistance of strains against human serum. The serum killing assays were performed by incubating three ST627-KL8 isolates, *K. pneumoniae* ATCC 43816 and *K. pneumoniae* ATCC 700603, with human serum for 1, 2, or 3 h at 37°C. Data are presented as mean ± SD derived from three separate experiments. One-way ANOVA with Dunnett’s test was used to analyze data, ****p < 0.0001. For panel **(C)**, ****p < 0.0001 and ***p < 0.001 indicated ST627-KL8 versus *K. pneumoniae* ATCC 43816, ^####^*p* < 0.0001 and ^###^*p* < 0.001 for ST627-KL8 versus *K. pneumoniae* ATCC 700603.

**FIGURE 6:**
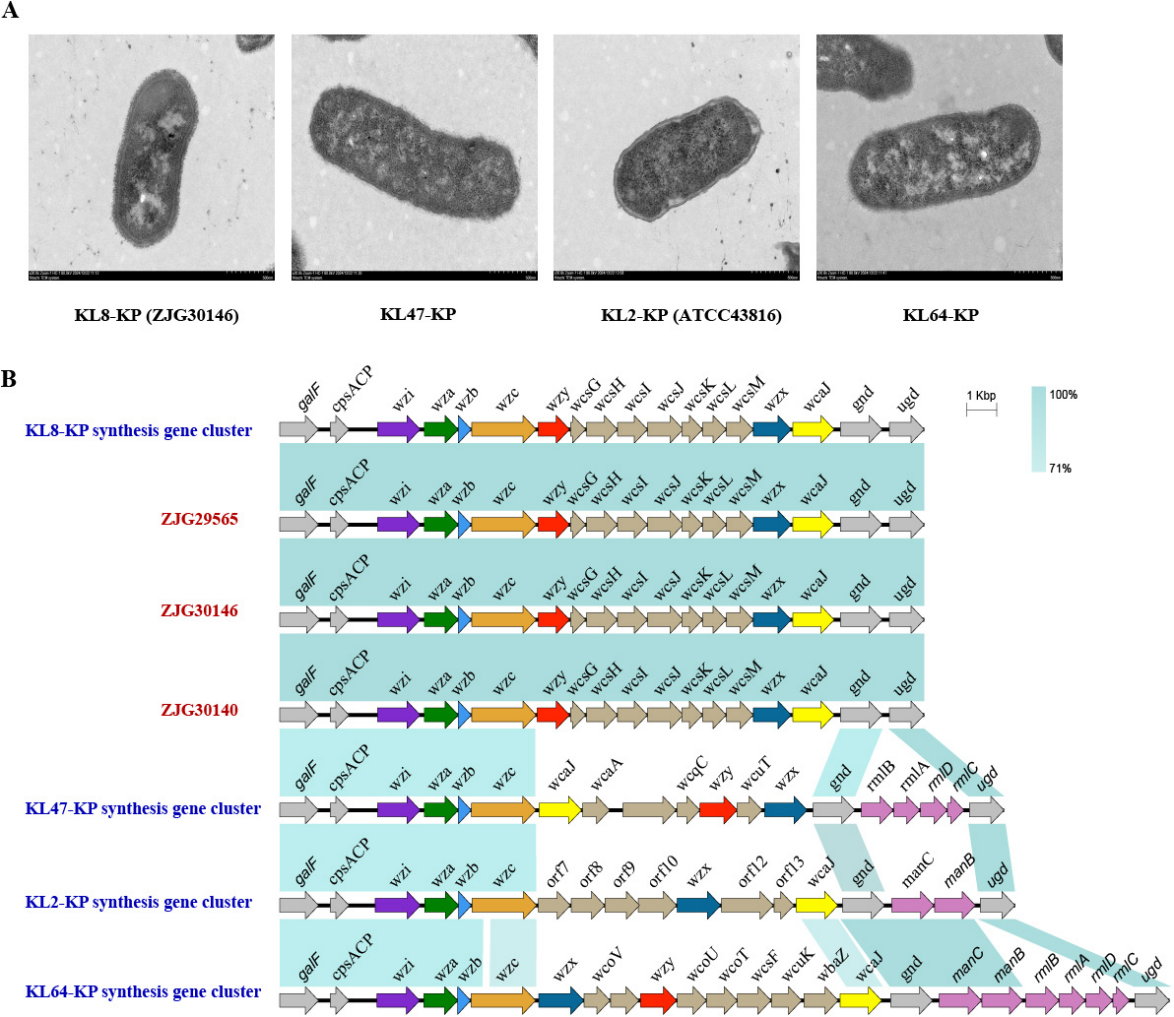
A distinct CPS gene cluster and a thinner capsule in ST627-KL8 CRKP. **(A)** Capsular morphology of KL8-KP (ZJG30146), KL47-KP, KL2-KP (*K. pneumoniae* ATCC 43816) and KL64-KP. KL47-KP and KL64-KP were also isolated from clinical patients and were stored in a previous study. *K. pneumoniae* ATCC 43816 was a KL2 serotype strain and was stored in our laboratory. **(B)** Genetic variations in *cps* gene clusters of three KL8 strains, KL2-KP, KL47-KP, and KL64-KP. The gene clusters of KL8-KP, KL2-KP, KL47-KP, and KL64-KP strains are obtained from the Kaptive Database. The gene cluster information of three isolates, including ZJG29565, ZJG30140, and ZJG30146, is provided by WGS analysis in this study.

In consideration of three isolates possessing an uncommon KL8 capsular type, as well as some K-types, such as KL1 and KL2, which were strongly correlated with virulence, we further characterized their capsule-associated phenotypes and genetic features. String test and semi-quantitative viscosity assays revealed that ST627-KL8 isolates exhibited lower mucoviscosity (Figure 5A) and significantly lower uronic acid content (Figure 5B) compared with *K. pneumoniae* ATCC 43816. In serum killing assays, the three isolates demonstrated intermediate serum resistance between the low-virulence strain *K. pneumoniae* ATCC 700603 and the hypervirulent strain *K. pneumoniae* ATCC 43816 (Figure 5C). Transmission electron microscopy (TEM) further confirmed that ST627-KL8 isolates possessed a thinner capsule compared to KL2, similar to that of KL47 and KL64 strains (Figure 6A). To explore the genomic basis of these phenotypes, we compared the capsular polysaccharide (CPS) gene clusters of KL8 with representative KL2, KL47, and KL64 strains using Easyfig v2.2.3 (Figure 6B). As expected, all *cps* loci shared a conserved region involved in capsule assembly, while the variable region between *wzy* and *wcaJ* differed substantially among K-types. The KL8 *cps* locus displayed the characteristic organization of this serotype, including a distinct set of *wcs* glycosyltransferase genes (e.g., *wcsG*, *wcsH*, *wcsI*) responsible for defining KL8-specific capsule structures. Importantly, Kaptive analysis showed that the KL8 *cps* locus in all three isolates was complete, with no missing or truncated genes, consistent with the observed low-capsule phenotype arising from intrinsic KL8 biosynthetic features rather than *cps* disruption.

### 3.6 Virulence in the *G. mellonella* infection model

**FIGURE 7:**
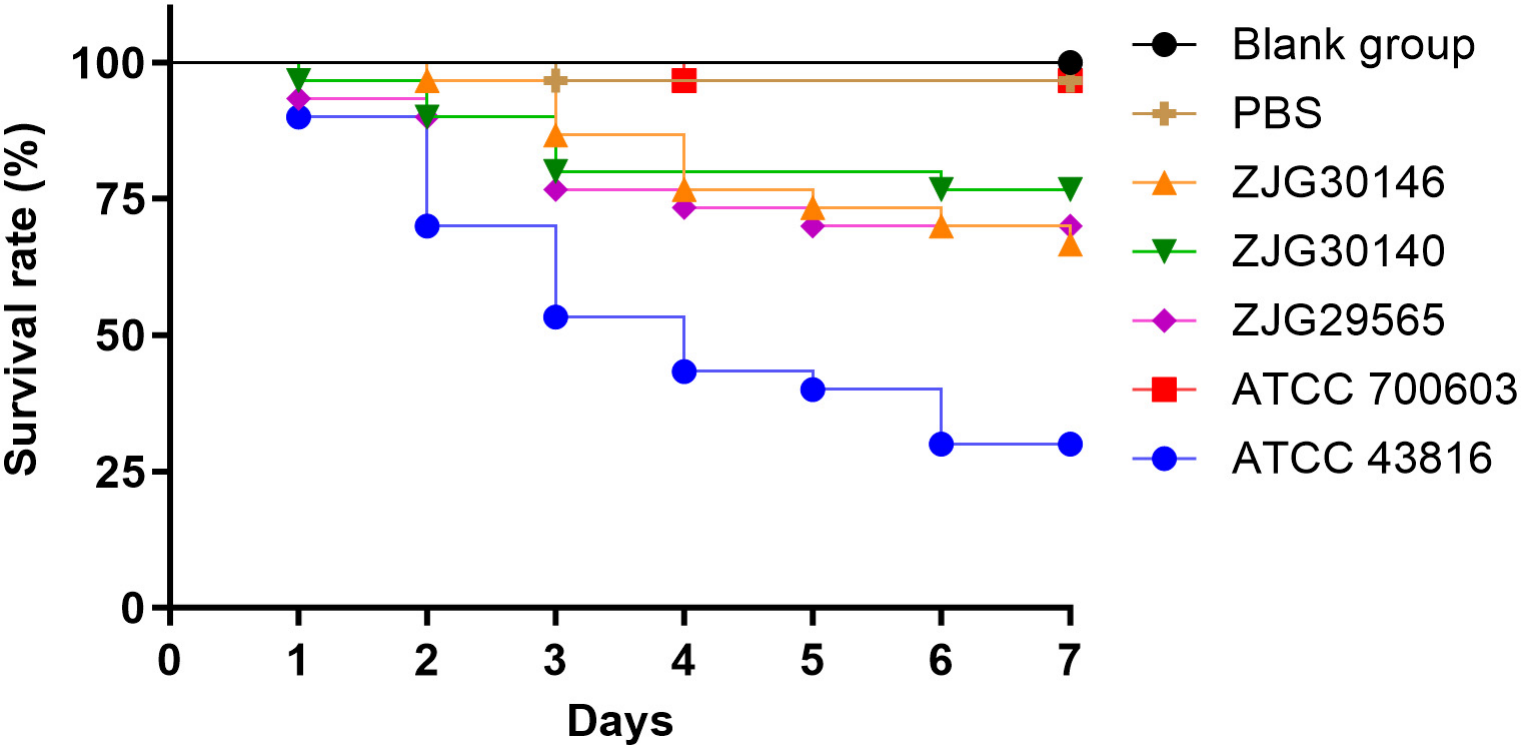
Virulence assessment of three ST627-KL8 CRKP isolates in the *G. mellonella* infection model. Larvae were injected with PBS (negative control), reference strains *K. pneumoniae* ATCC 700603 (low-virulence control), *K. pneumoniae* ATCC 43816 (hypervirulent control), and three ST627-KL8 CRKP isolates (ZJG29565, ZJG30140, ZJG30146) separately. Survival was monitored over time (*n* = 30 larvae per group). Survival curves were generated using the Kaplan–Meier method, and statistical significance between groups was determined by the Log-rank test. Data are representative of three independent experiments.

We utilized a *G. mellonella* infection model to evaluate the virulence of ST627-KL8 isolates. The virulence assessment revealed notable disparities in larval survival among the experimental groups. The low-virulence strain *K. pneumoniae* ATCC 700603 exhibited a high survival rate of 96.7% (29/30 larvae), statistically comparable to that of the PBS control group (96.7%, 29/30). Conversely, the hypervirulent strain *K. pneumoniae* ATCC 43816 showed a significantly reduced survival rate of 30.0% (9/30). The clinical isolates displayed intermediate virulence phenotypes, with survival rates of 66.7% (20/30), 76.7% (23/30), and 70.0% (21/30) for ZJG30146, ZJG30140, and ZJG29565, respectively (Figure 7).

## 4 Discussion

Our study unveils a localized, small-scale outbreak involving three unusual ST627-KL8 CRKP strains within an ICU ward of a tertiary hospital. WGS analysis revealed that three isolates exhibited ≤ 5 SNP differences, strongly supporting a single transmission chain (Jia and Ruan, 2022). This retrospective analysis, based on the isolation of three ST627-KL8 CRKP isolates over a 46-day period (April to June 2018) within the ICU, reveals a novel serotype lineage of *K. pneumoniae* in China. The emergence of this novel lineage may reflect selection pressures in the ICU environment, particularly the intense antibiotic use. This observation highlights the critical need for enhanced vigilance, strengthened surveillance, and in-depth analyses of its evolutionary trajectory to mitigate future dissemination and reduce its potential clinical impact.

*In vitro*, the three ST627-KL8 strains exhibited a hypermucoviscous phenotype with low uronic acid content, along with moderate serum resistance. Furthermore, *in vivo* infection assays using the *G. mellonella* model confirmed that ST627-KL8 CRKP isolates in this study were not hypervirulent. Consistently, genomic analysis revealed the absence of classical hypervirulence genes in all three ST627-KL8 strains.

Comparative analysis of the KL8 capsular polysaccharide (*cps*) locus demonstrated that all three ST627-KL8 isolates possessed a structurally complete KL8 region, with full gene content and no missing or truncated elements detected by Kaptive. Unlike hypervirulent K-types such as KL1 and KL2, which contain expanded glycan-modifying enzymes and accessory genes that promote thick capsule formation and hypermucoviscosity, the KL8 biosynthetic region is inherently more streamlined. Although the *wcs*/*wca*-associated glycosyltransferases required for initiating polysaccharide synthesis are present, KL8 lacks the additional modification machinery that contributes to the highly mucoid phenotype characteristic of hvKP. This genomic architecture is consistent with the reduced uronic acid content, thin capsule ultrastructure, and hypermucoviscous phenotype observed in our strains, suggesting that the intermediate virulence of ST627-KL8 is largely attributable to the intrinsic properties of the KL8 *cps* locus rather than *cps* disruption or acquisition of hypervirulence determinants. However, a discrepancy exists between these model results and the clinical presentations. Notably, the positive outcome for Patient 2 can likely be attributed to several factors: young age (25-year-old), absence of underlying comorbidities, a relatively robust immune system, timely identification of the CRKP strain, and subsequent administration of tigecycline. Conversely, the other two patients were elderly individuals with pre-existing conditions. The subtle clinical manifestations associated with this low-virulence strain may have contributed to delayed recognition and delayed initiation of appropriate antimicrobial therapy, particularly in patients with poor prognostic factors.

It is important to note that although two patients were discharged against medical advice (DAMA), these decisions primarily reflected complex clinical and family considerations—such as severe comorbidities, limited perceived benefit of further aggressive treatment, and local end-of-life care preferences—rather than pathogen-driven mortality. Therefore, DAMA should not be interpreted as evidence of an adverse clinical outcome directly attributable to the ST627-KL8 strain.

Each of the three clinical isolates harbored an IncFII_K34_ plasmid carrying the *bla*_KPC–2_ gene. IncFII_K34_ plasmids have been reported to possess markedly higher conjugation frequencies—100 to 1,000-fold higher than that of the prevalent IncFII_K2_ KPC-2 plasmid—due to overexpression of conjugation-related genes and increased *bla*_KPC_ copy number and expression (Jiang et al., 2025). The prevalence of invasive procedures, such as mechanical ventilation, among ICU patients, coupled with the efficient horizontal transfer capability of plasmids like IncFII_K34_, significantly increases opportunities for the dissemination of resistant bacteria. This presents a major challenge in the ICU environment, where antibiotic usage, particularly carbapenems like meropenem, is intensive. Although the IncFII_K34_ plasmid identified in this study lacked classic virulence genes, its presence carrying *bla*_KPC–2_ in the ST627-KL8 isolates still warrants attention. However, we did not experimentally assess the conjugative transfer of this IncFII_K34_ plasmid or characterize potential transconjugants, which represents an additional limitation of our study and should be addressed in future work.

A limitation of our retrospective study is the small sample size, with only three ST627-KL8 CRKP isolates obtained through retrospective sampling. This constrained our capacity to investigate the origin and disappearance of this novel lineage in the ICU ward. This finding highlights the need for timely and comprehensive surveillance incorporating both clinical and environmental sampling to track the dissemination of this specific *K. pneumoniae* lineage. Nevertheless, even this limited outbreak involving a rare clone provided valuable insights and prompted critical considerations for further investigation. Recent genomic studies suggest that specific uncommon capsular variants may confer unexpected ecological advantages in particular niches or infection contexts, potentially facilitating the emergence of novel pathogenic lineages (Wyres et al., 2020).

## Data Availability

The sequencing data are available under BioProject PRJNA1291675. The genome assemblies are available under GenBank assembly accessions GCF_053322435.1, GCF_051590465.1, and GCF_051590525.1.
