# Supplementary material for: Emergence and characterization of a novel ST627-KL8 carbapenem-resistant Klebsiella pneumoniae lineage associated with ICU transmission in a tertiary hospital, China
Source: Front Microbiol. 2026 Feb 4;16:1723336. doi: 10.3389/fmicb.2025.1723336 (PMC12915689; doi:10.3389/fmicb.2025.1723336)
Supplement: Supplementary file 1 [file Data_Sheet_1.pdf]

Supplemental Figure 1

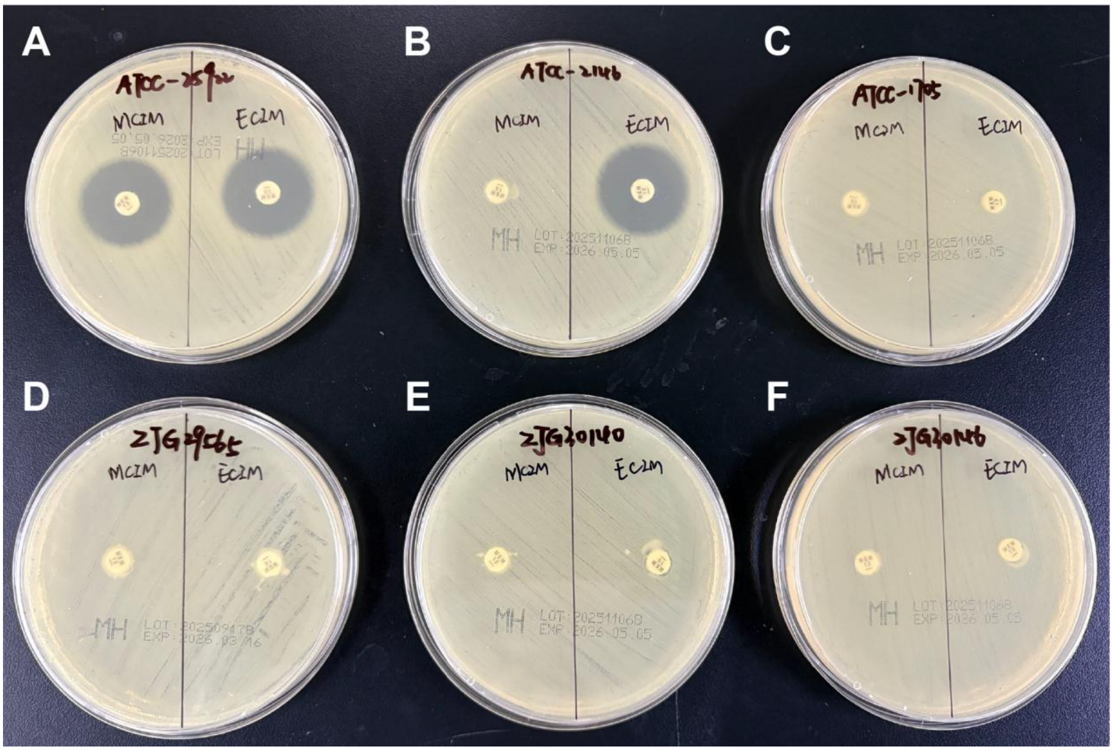

Supplemental Figure 1. mCIM and eCIM results for the three ST627-KL8 CRKP isolates. (D-F) All three isolates (ZJG29565, ZJG30140, ZJG30146) showed mCIM-positive and eCIM-negative phenotypes, consistent with KPC-type serine carbapenemase production. (C) *K. pneumoniae* ATCC BAA-1705 (KPC producer) and (B) *K. pneumoniae* ATCC BAA-2146 (NDM producer) served as positive controls; (A) *E. coli* ATCC 25922 was used as the negative control.

Supplemental Figure 2

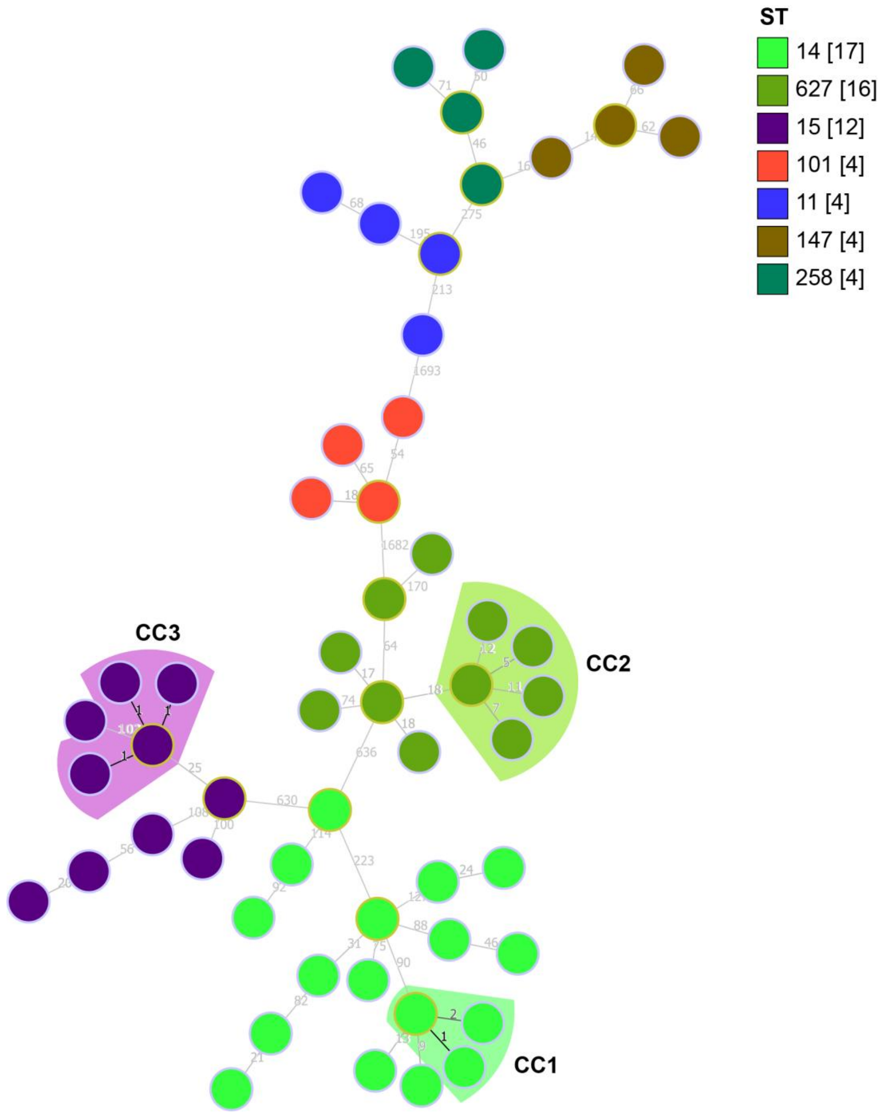

Supplemental Figure 2. Core-genome MLST minimum spanning tree of ST627 and related *K. pneumoniae* sequence types. A minimum spanning tree was generated in Ridom SeqSphere+ using the K. pneumoniae cgMLST scheme for 61 isolates, including ST627, ST14, ST15 and representative high-risk clones (ST11, ST101, ST147 and ST258). Each circle represents one isolate, and node colours indicate sequence type (ST) as shown in the legend (light green, ST14; dark green, ST627; purple, ST15; red, ST101; blue, ST11; brown, ST147; teal, ST258). Numbers on the connecting lines denote allelic distances between neighbouring nodes. Shaded regions (CC1-CC3) highlight clonal complexes defined in the SeqSphere+ scheme by ≤15 allelic differences and correspond to subsets of ST14 (CC1), ST15 (CC2) and ST627 (CC3); additional isolates of these STs lie outside the shaded areas because they exceed this threshold. The close positioning of the ST627 cluster (CC3) adjacent to the ST14 cluster (CC1) illustrates that ST627 is allelically most closely related to ST14 among the sequence types analysed.
