# Supplementary material for: Emergence and characterization of a novel ST627-KL8 carbapenem-resistant Klebsiella pneumoniae lineage associated with ICU transmission in a tertiary hospital, China
Source: Front Microbiol. 2026 Feb 4;16:1723336. doi: 10.3389/fmicb.2025.1723336 (PMC12915689; doi:10.3389/fmicb.2025.1723336)
Supplement: Supplementary file 2 [file Table_1.docx]

**Supplementary Table 1. SNP differences among three ST627-KL8 CRKP isolates**

| **CHR^a^** | **POS^b^** | **ZJG30146（REF^c^）** | **ZJG30140** | **ZJG29565** | **FTYPE^d^** | **STRAND** | **NT_POS^e^** | **AA_POS^f^** |
| --- | --- | --- | --- | --- | --- | --- | --- | --- |
| Chrom1 | 1824455 | A | A | C |  |  |  |  |
| Chrom1 | 2319236 | A | A | G | CDS | + | 466/825 | 156/274 |
| Chrom1 | 2619965 | A | A | T | CDS | - | 9/1272 | 3/423 |
| Chrom1 | 2885230 | G | G | C | CDS | + | 408/924 | 136/307 |
| Chrom1 | 3640279 | T | T | G | CDS | - | 645/1227 | 215/408 |

^a^ CHR: Chromosome

^b^ POS: Position in chromosome sequence

^c^ REF: Reference strain

^d^ FTYPE: Mutation type

^e^ NT_POS: Nucleotide position in CDS

^f^ AA_POS: Amino acid position in CDS
