# Supplementary material for: Emergence and characterization of a novel ST627-KL8 carbapenem-resistant Klebsiella pneumoniae lineage associated with ICU transmission in a tertiary hospital, China
Source: Front Microbiol. 2026 Feb 4;16:1723336. doi: 10.3389/fmicb.2025.1723336 (PMC12915689; doi:10.3389/fmicb.2025.1723336)
Supplement: Supplementary file 3 [file Table_2.docx]

**Supplementary Table 2. Genomic information of nine reference strains compared to ST627-KL8 isolates.**

| **Description** | **Max Score** | **Total Score** | **Query Cover** | **E value** | **Per. Ident^a^** | **Acc. Len^b^** | **Accession** |
| --- | --- | --- | --- | --- | --- | --- | --- |
| *Klebsiella pneumoniae* strain U25 genome | 8.33E+05 | 1.06E+07 | 98% | 0 | 99.91% | 5491870 | CP012043.1 |
| *Klebsiella pneumoniae* strain NKP9 chromosome | 6.70E+05 | 9.36E+06 | 95% | 0 | 99.66% | 5377832 | CP091827.1 |
| *Klebsiella pneumoniae* strain FDAARGOS_1308 chromosome | 8.33E+05 | 1.04E+07 | 97% | 0 | 99.91% | 5303185 | CP069968.1 |
| *Klebsiella pneumoniae* strain B31 chromosome | 8.78E+05 | 1.02E+07 | 97% | 0 | 99.85% | 5273146 | CP035929.1 |
| *Klebsiella pneumoniae* strain 19051 chromosome | 3.82E+05 | 1.01E+07 | 96% | 0 | 99.93% | 5332976 | CP022023.1 |
| *Klebsiella pneumoniae* strain DA12090 chromosome | 8.17E+05 | 1.04E+07 | 96% | 0 | 99.87% | 5311952 | CP030072.1 |
| *Klebsiella pneumoniae* strain 12 chromosome | 7.73E+05 | 1.05E+07 | 97% | 0 | 99.69% | 5346226 | CP097220.1 |
| *Klebsiella pneumoniae* strain DEUKp6718 chromosome | 3.66E+05 | 9.69E+06 | 92% | 0 | 99.87% | 5171309 | CP113839.1 |
| *Klebsiella pneumoniae* strain F4 chromosome | 8.42E+05 | 1.01E+07 | 96% | 0 | 99.86% | 5249104 | CP090397.1 |

^a^ Per. Ident：Percent Identity

^b^ Acc. Len：Accession Length
