# Supplementary material for: Emergence and characterization of a novel ST627-KL8 carbapenem-resistant Klebsiella pneumoniae lineage associated with ICU transmission in a tertiary hospital, China
Source: Front Microbiol. 2026 Feb 4;16:1723336. doi: 10.3389/fmicb.2025.1723336 (PMC12915689; doi:10.3389/fmicb.2025.1723336)
Supplement: Supplementary file 4 [file Table_3.docx]

**Supplementary Table 3. Strain list for phylogenetic tree construction.**

| **Biosample** | **Assembly** | **GenBank** | **Sample name** | **ST** | **KL type** | **O type** | **KPC-2** |
| --- | --- | --- | --- | --- | --- | --- | --- |
| SAMEA12944458 | PDT001385184.1 | GCA_025044015.1 | JPI280_S70 | 627 | KL24 | O1 | - |
| SAMEA114492533 | PDT001958031.1 | GCA_033123245.1 | KP_NORM_URN_109001 | 627 | KL24 | O1 | - |
| SAMEA110371227 | - | - | Kp_4249 | 627 | KL24 | O1 | - |
| SAMEA11291424 | - | - | MVK-11F265 | 627 | KL24 | O1 | - |
| SAMEA5049811 | - | - | SPARK_2608_C1 | 627 | KL24 | O1 | - |
| SAMEA8948709 | PDT001092075.1 | GCA_021921905.1 | KP_NORM_BLD_2014_104360 | 627 | KL24 | O1 | - |
| SAMN15233234 | PDT000907925.1 | GCA_046198945.1 | K75 | 627 | KL24 | O1 | - |
| SAMN15233235 | PDT000907924.1 | GCA_046199095.1 | K90 | 627 | KL24 | O1 | - |
| SAMN15233232 | PDT000907923.1 | GCA_046199035.1 | K04 | 627 | KL24 | O1 | - |
| SAMN12731936 | PDT000628217.1 | GCA_022212785.1 | N16-03101 | 627 | KL24 | O1 | - |
| SAMEA4427690 | - | - | SCP24-25 | 627 | KL24 | O1 | - |
| SAMN15233233 | PDT000907922.1 | GCA_046199075.1 | K69 | 627 | KL24 | O1 | - |
| SAMN14898741 | ASM1318761v1 | GCA_013187615.1 | L18 | 627 | KL54 | O1 | - |
| SAMN16093053 | ASM3343411v1 | GCA_033434115.1 | 09Q348 | 15 | KL64 | O1 | - |
| SAMN13031516 | ASM920793v1 | GCA_009207935.1 | 071I10 | 14 | unknown (KL2) | O2a | - |
| SAMN02581368 | Kleb_pneu_CHS_66_V1 | GCA_000694235.1 | CHS 66 | 14 | KL2 | O1 | - |
| SAMEA3512030 | 18174_4#2 | GCA_900512305.1 | EuSCAPE_DE003 | 14 | KL2 | O1 | - |
| SAMN15421726 | ASM1340170v1 | GCA_013401705.1 | UPMP2118 | 15 | KL24 | O1 | - |
| SAMN19374566 | ASM1879083v1 | GCA_018790835.1 | UPMP 1161 | 15 | KL24 | O1 | - |
| SAMN03780451 | ASM146316v1 | GCF_001463165.1 | U25 | 14 | KL2 | O1 | - |
| SAMEA3531878 | 18090_8#75 | GCA_900093275.1 | W2-4-ERG4 | 15 | KL24 | O1 | - |
| SAMN19374571 | ASM1879004v1 | GCA_018790045.1 | UPMP 1160 | 15 | KL24 | O1 | - |
| SAMN19374569 | ASM1879020v1 | GCA_018790205.1 | UPMP 2091 | 15 | KL24 | O1 | - |
| SAMN13563923 | ASM1940116v1 | GCA_019401165.1 | K509 | 14 | KL2 | O1 | - |
| SAMN13563919 | ASM1940126v1 | GCA_019401265.1 | K439 | 14 | KL2 | O1 | - |
| SAMN21016514 | ASM2113648v1 | GCA_021136485.1 | BD-2 | 15 | KL24 | O1 | - |
| SAMN24667609 | ASM2153050v1 | GCA_021530505.1 | 108 | 14 | KL2 | O1 | - |
| SAMN16093059 | ASM3343403v1 | GCA_033434035.1 | 13T155 | 15 | KL64 | O1 | - |
| SAMEA3721131 | 19084_4#110 | GCA_900505005.1 | EuSCAPE_HR101 | 14 | KL2 | O1 | - |
| SAMEA3729879 | 19646_3#103 | GCA_900508155.1 | EuSCAPE_TR282 | 14 | KL2 | O1 | - |
| SAMN15898402 | ASM1676249v1 | GCA_016762495.1 | GCKp10 | 14 | KL16 | O1 | - |
| SAMN16357450 | ASM1690379v1 | GCA_016903795.1 | FDAARGOS_1308 | 14 | KL2 | O1 | - |
| SAMN19374577 | ASM1879078v1 | GCA_018790785.1 | UPMP 2046 | 15 | KL24 | O1 | - |
| SAMEA3721094 | 19084_4#75 | GCA_900506075.1 | EuSCAPE_HR062 | 14 | KL2 | O1 | - |
| SAMN19374580 | ASM1878999v1 | GCA_018789995.1 | UPMP 2089 | 15 | KL24 | O1 | - |
| SAMN32525362 | ASM2907818v1 | GCA_029078185.1 | PS_Kpn28 | 14 | KL2 | O1 | - |
| SAMN07513381 | ASM293474v1 | GCA_002934745.1 | SKLX003155 | 14 | KL16 | O1 | - |
| SAMN13563924 | ASM1940120v1 | GCA_019401205.1 | K515 | 14 | KL2 | O1 | - |
| SAMN20369324 | ASM2539745v1 | GCA_025397455.1 | stjyk0001 | 15 | unknown (KL24) | O1 | - |
| SAMN05188406 | ASM166319v1 | GCA_001663195.1 | BR | 15 | KL112 | O1 | + |
| SAMN11579543 | ASM1810837v1 | GCA_018108375.1 | CRE239 | 14 | KL16 | O1 | - |
| SAMN21016522 | ASM2113696v1 | GCA_021136965.1 | 6507.79 | 14 | KL2 | O1 | - |
| SAMNO2602959 | PDT000007400.2 | GCA 000240185.2 | HS11286 | 11 | KL103 | O2a | + |
| SAMNO9487517 | PDT000510487.1 | GCA 005854245.1 | CR-HVKP4 | 11 | unknown | unknown | + |
| SAMN16205030 | PDTO02714663.2 | GCA 049532805.1 | CRKP-Urine1 | 11 | KL64 | O2a | + |
| SAMNO2152539 | PDTO00022644.2 | GCA 000364385.3 | ATCC BAA-2146 | 11 | unknown | O2ac | - |
| SAMNO3081501 | PDT000024213.1 | GCA 000597905.1 | 30684/NJST258 2 | 258 | KL107 | O2afg | - |
| SAMN03081502 |  | GCA 000598005.1 | 30660/NJST258 1 | 258 | KL107 | O2afg | - |
| SAMN01057611 | PDT000009640.3 | GCA 000281535.2 | KPNIH1 | 258 | KL107 | unknown | - |
| SAMNO2786855 | PDT000034574.1 | GCA 000714675.1 | KPNIH24 | 258 | KL106 | O2afg | + |
| SAMN15946737 | PDT000834374.1 | GCA 014495765.1 | HKP0067 | 147 | unknown | O2a | - |
| SAMN07312483 | PDT000253696.1 | GCA 002591075.1 | FDAARGOS 439 | 147 | KL64 | O2ac | - |
| SAMNO8026708 | PDT000304317.1 | GCA 003031345.1 | NH54 | 147 | KL10 | O3 | - |
| SAMN15676882 | PDT000984757.1 | GCA 017310425.1 | IR5086 | 147 | KL81 | unknown | - |
| SAMN39589781 | PDT002071991.2 | GCA 036347655.1 | B18185 | 101 | KL17 | O1 | - |
| SAMNO5412455 | PDT0001718511 | GCA 001902435.1 | Kp_Goe_33208 | 101 | KL17 | O1 | - |
| SAMEA115720014 | PDT0022509511 | GCA964198855.1 | Zagreb011 | 101 | KL17 | O1 | - |
| SAMN21016516 | PDT001201675.1 | GCA021135615.1 | BD-41 | 101 | KL17 | O1 | - |
