# Supplementary material for: Emergence and characterization of a novel ST627-KL8 carbapenem-resistant Klebsiella pneumoniae lineage associated with ICU transmission in a tertiary hospital, China
Source: Front Microbiol. 2026 Feb 4;16:1723336. doi: 10.3389/fmicb.2025.1723336 (PMC12915689; doi:10.3389/fmicb.2025.1723336)
Supplement: Supplementary file 5 [file Table_4.docx]

**Supplementary Table 4. Genomic information of nine reference plasmids compared to the plasmid pZJG29565.**

| **Description** | **Max Score** | **Total Score** | **Query Cover** | **E value** | **Per. Ident^a^** | **Acc. Len^b^** | **Accession** |
| --- | --- | --- | --- | --- | --- | --- | --- |
| *Klebsiella pneumoniae* subsp. *pneumoniae* HS11286 plasmid pKPHS2 | 54222 | 1.87E+05 | 90% | 0 | 98.08% | 111195 | CP003224.1 |
| *Escherichia coli* strain Ecol_AZ153 plasmid pECAZ153_KPC | 56244 | 1.93E+05 | 84% | 0 | 99.23% | 146162 | CP018999.1 |
| *Klebsiella pneumoniae* strain AR_0139 plasmid tig00000003 | 35809 | 1.39E+05 | 67% | 0 | 97.82% | 89382 | CP021959.1 |
| *Klebsiella pneumoniae* strain KF3 plasmid pKF3-94 | 37349 | 1.42E+05 | 68% | 0 | 99.16% | 94219 | FJ876826.1 |
| *Klebsiella pneumoniae* subsp. pneumoniae strain NUHL24835 plasmid | 37358 | 1.45E+05 | 69% | 0 | 99.17% | 101030 | CP014005.1 |
| *Klebsiella pneumoniae* strain KPN1482 plasmid pKPN1482-2 | 35798 | 1.48E+05 | 64% | 0 | 97.81% | 97202 | CP020843.1 |
| *Klebsiella pneumoniae* strain KP048 plasmid pKP048 | 37338 | 1.50E+05 | 76% | 0 | 99.33% | 151188 | FJ628167.2 |
| *Klebsiella pneumoniae* isolate Kp_Goe_154414 plasmid pKp_Goe_414-3 | 38082 | 1.21E+05 | 58% | 0 | 99.79% | 81939 | CP018340.1 |
| *Klebsiella pneumoniae* strain KP36 plasmid 1 | 1.06E+05 | 1.61E+05 | 74% | 0 | 99.90% | 155781 | CP017386.1 |

^a^ Per. Ident：Percent Identity

^b^ Acc. Len：Accession Length
